# Ankle dorsiflexion deficit in the back leg is a risk factor for shoulder and elbow injuries in young baseball players

**DOI:** 10.1038/s41598-021-85079-8

**Published:** 2021-03-09

**Authors:** Hitoshi Shitara, Tsuyoshi Tajika, Takuro Kuboi, Tsuyoshi Ichinose, Tsuyoshi Sasaki, Noritaka Hamano, Takafumi Endo, Masataka Kamiyama, Akira Honda, Ryosuke Miyamoto, Kurumi Nakase, Atsushi Yamamoto, Tsutomu Kobayashi, Kenji Takagishi, Hirotaka Chikuda

**Affiliations:** Department of Orthopedic Surgery, Gunma University Graduate School of Medicine, 3-39-22, Showa, Maebashi, Gunma 371-8511 Japan

**Keywords:** Risk factors, Orthopaedics

## Abstract

The relationship between ankle joint function and throwing-related injuries has not been demonstrated. We hypothesized that limited ankle joint range of motion (ROM) was related to risk factors for shoulder and elbow injuries in young baseball players. This 12-month prospective cohort study evaluated the age, height, weight, playing position, shoulder, elbow, and ankle function of 228 enrolled baseball players. Shoulder and elbow injuries were tracked during the season. Univariate and multivariate analyses were performed to identify risk factors for shoulder and elbow injuries among participants divided into non-injured and injured groups. Univariate analysis showed that age, height, weight, ROM of elbow flexion in the dominant arm, muscle strength ratio of shoulder abduction, and the likelihood of being a pitcher or a catcher were significantly greater in the injured group than in the non-injured group. ROM of shoulder abduction-external/internal rotation, shoulder total arc on the dominant arm, ankle joint dorsiflexion, and plantar flexion on the back (non-lead) and front (lead) legs were significantly less in the injured group than in the non-injured group. In conclusion, ROM dorsiflexion deficits in the back leg, shoulder abduction-external rotation in the dominant arm, ROM increase in elbow flexion on the dominant side, older age, and being a pitcher were significant independent risk factors for injury.

## Introduction

Shoulder and elbow pain and injuries are major issues among baseball players^1–3^. Throwing involves a whole-body movement that starts from the lower limbs with motion transmitted to the upper limbs via the trunk. Therefore, maintaining sufficient function of the whole body related to throwing is necessary to maintain optimal performance and prevent shoulder and elbow injuries.

Previous studies have reported that risk factors for shoulder and elbow pain and injuries involve a decreased range of motion (ROM) in the dominant shoulder, shoulder external rotation insufficiency, preseason total shoulder rotation deficit, preseason supraspinatus^4^, and prone external rotation strength deficits^4–8^. While a few studies have investigated the risk factors for shoulder and elbow injuries related to dysfunction of the trunk and lower extremities, throwing-related shoulder and elbow injuries are reportedly significantly associated with trunk and lower limb dysfunction, involving hip joint ROM deficits, abnormal foot posture, lack of lumbopelvic control, and inadequate dynamic balance^8–21^. However, studies on the relationship between ankle joint function and throwing-related injuries are lacking, despite adequate ankle joint function being essential to provide and transmit kinetic energy to the shoulder and elbow^22,23^.

Based on the relationship between baseball performance and upper and lower extremity kinematics, Kung et al.^24^ investigated whether adolescent baseball pitchers experience changes in ball speeds and lower extremity kinematics during the stride phase between the 1–15 fastball pitches (baseline set) and 76–90 fastball pitches (final set). The study showed a significant decrease in the hip extension (baseline: 14.7° ± 9.8°; final: 11.6° ± 10.3°; P < 0.05) and the ankle plantar flexion (baseline: 30.2° ± 14.5°; final: 24.2° ± 15.3°; P < 0.05) on the back leg as well as ball speeds (baseline: 29.5 ± 2.5 m/s; final: 28.3 ± 2.5 m/s; P < 0.05) during final set compared with during baseline set. Gdovin et al.^25^ suggested that the footwear worn by a youth baseball pitcher altered the shoulder and elbow dynamics in the dominant throwing arm as well as the amount of ankle plantarflexion in the stride leg.

Based on the predicting risk factors for sports injury from the physical conditioning evaluated by the Functional Movement Screen (FMS)^26,27^, which has been developed to detect abnormal functional movement patterns, Liang et al.^28^ suggested that the FMS may have a role in predicting athletic performance instead of injury because the FMS score was not significantly associated with core stability, muscular strength, or muscle flexibility in elite collegiate baseball players. In high school baseball players, Lee et al.^29^ reported that utilizing the FMS for injury prediction is not recommended because of low sensitivity. Thus, to our knowledge, this is the first prospective study to investigate this relationship. Therefore, we aimed to prospectively investigate whether ankle joint ROM affected the incidence of shoulder and elbow injuries in young baseball players after adjusting for differences in demographic data and upper extremity function.

## Results

### Participants

**Figure 1:**
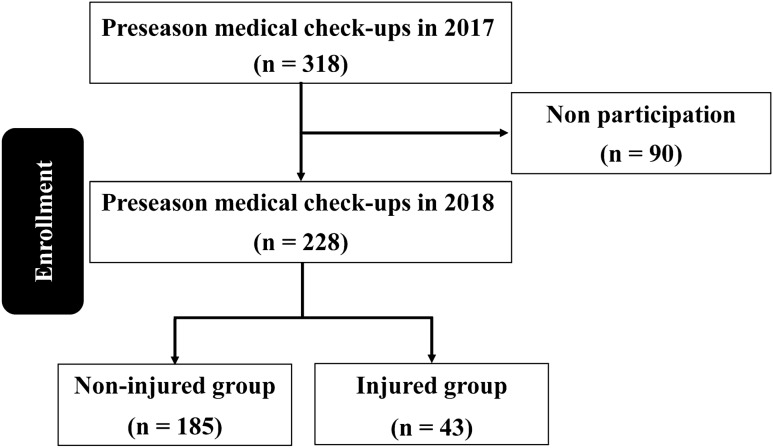
Flow chart of the players included in this study.

In total, 318 players participated in a preseason medical check-up in 2017, and of these, 228 who also participated in a preseason medical check-up in 2018 were enrolled in this study. The incidence of injury was 18.8% (n = 43: shoulder, 7; elbow, 32; both shoulder and elbow, 4; Fig. 1).

### Baseline characteristics

To identify and analyze variations between injured and non-injured players, participants were classified into injured and non-injured groups. Age, height, and weight were significantly greater in the injured group than in the non-injured group. Chi-square tests showed that playing position significantly differed between the groups (P = 0.001). A subsequent residual analysis revealed that the percentage of participants who were fielders was significantly larger in the non-injured group than in the injured group, while the percentage of participants who were pitchers or catchers was significantly larger in the injured group than in the non-injured group (Table 1).

**Table 1:** Baseline characteristics of the study participants.

Baseline characteristics	Non-injured (n = 185)		Injured (n = 43)		*P* value	
	Mean	SEMc6	Mean	SEM		
Age (year)	10.2	0.1	11.0	0.0	0.000	*
Height (cm)	139.7	0.7	146.8	1.4	0.000	*
Weight (kg)	33.9	0.6	40.4	1.3	0.000	*

	N (adjusted residual)	N (adjusted residual)				
**Position**			0.001	*		
Fielder	153 (3.8)	24 (− 3.8)		*		
Catcher	18 (− 2.0)	9 (2.0)		*		
Pitcher	14 (− 3.0)	10 (3.0)		*		

*SEM* standard error of the mean.

*P < 0.05.

### Univariate analysis

Univariate analyses showed that elbow flexion ROM and supraspinatus strength on the dominant side were significantly greater in the injured group than in the non-injured group. 90°-abducted external and internal rotation shoulder joint ROMs on the dominant side, shoulder total arc on the dominant side, ankle dorsiflexion and plantarflexion on the back leg, and plantarflexion on the front leg were significantly smaller in the injured group than in the non-injured group. While there were no significant differences between the groups in terms of elbow extension ROM and shoulder horizontal adduction on the dominant side, differences were observed in 90°-abducted external and internal rotation, total arc, and horizontal adduction; supraspinatus, prone external rotation, and prone internal rotation muscle strength on the dominant side; prone external rotation and prone internal rotation strength ratios; and ankle dorsiflexion on the front leg (Table 2).

**Table 2:** Results of univariate analyses.

Univariate analysis	Non-injured (n = 185)		Injured (n = 43)		*P* value	
	Mean	SEM	Mean	SEM		
**Elbow ROM (deg)**						
Extension on dominant side	6.4	0.4	6.7	1.2	0.780	
Flexion on dominant side	139.2	0.5	141.9	0.8	0.011	*
**Shoulder ROM (deg)**						
ABER on dominant side	118.3	0.9	111.8	2.5	0.005	*
Difference in ABER	7.1	0.9	6.0	1.7	0.571	
ABIR on dominant side	47.4	1.0	42.6	1.8	0.035	*
Difference in ABIR	− 7.6	1.0	− 6.5	1.8	0.651	
Total arc on dominant side	165.7	1.5	154.4	3.3	0.001	*
Difference in total arc	− 0.5	1.2	− 0.5	2.1	0.979	
HF on dominant side	20.5	0.7	19.1	1.6	0.427	
Difference in HF	− 9.4	0.8	− 9.6	2.4	0.916	
**Shoulder strength**						
SS on dominant side (kg)	7.6	0.2	7.5	0.6	0.981	
SS ratio	1.0	0.0	1.1	0.0	0.005	*
PER on dominant side (kg)	11.8	0.3	11.0	0.8	0.321	
PER ratio	1.0	0.0	1.1	0.0	0.498	
PIR on dominant side (kg)	12.7	0.4	12.9	1.1	0.913	
PIR ratio	1.1	0.1	1.0	0.0	0.667	
PER/PIR ratio	1.0	0.0	0.9	0.0	0.113	
**Ankle ROM (deg)**						
Dorsal flexion on back leg	22.9	0.5	19.3	1.2	0.004	*
Dorsal flexion on front leg	23.4	0.6	21.0	1.2	0.065	
Plantar flexion on back leg	50.8	0.7	47.6	0.9	0.005	*
Plantar flexion on front leg	51.2	0.7	48.0	1.1	0.030	*

*SEM* standard error of the mean, *ROM* range of motion, *ABER and ABIR* ROM of 90°-abducted shoulder external and internal rotation, respectively, Total arc = ABER + ABIR, HA: horizontal adduction, Difference = ROM on dominant side—ROM on non-dominant side.

*SS*: seated supraspinatus, *PER*: prone external rotation, *PIR*: prone internal rotation.

Strength ratio = strength of the dominant side/strength of the non-dominant side.

PER/PIR strength ratio = PER strength of the dominant side/PIR strength of the dominant side

*P < 0.05.

### Multivariate analysis

Before performing a logistic regression analysis, Pearson’s correlation coefficient was calculated to avoid multicollinearity. Among the variables with a P value of < 0.1, based on the results of univariate analyses, height and weight significantly correlated with age (P < 0.001; correlation coefficient, 0.711 and 0.630, respectively), and shoulder total arc on the dominant side significantly correlated with 90°-abducted external and internal rotation (P < 0.001, correlation coefficient, 0.767 and 0.759, respectively). Therefore, height, weight, and shoulder total arc on the dominant side were excluded from the analyses. Logistic regression analysis showed that a ROM deficit in dorsiflexion in the back leg (odds ratio [OR] 0.944) and 90°-abducted shoulder external rotation on the dominant side (OR 0.970), greater elbow flexion ROM on the dominant side (OR 1.090), and older age (OR 1.994) were significant independent risk factors for shoulder and elbow injuries (Table 3).

**Table 3:** Results of multivariate analyses.

Multivariate analysis	Odds ratio	95% CI	*P* value	
Ankle dorsiflexion on back leg	0.944	0.896–0.994	0.028	*
Shoulder ABER on dominant side	0.970	0.945–0.996	0.022	*
Elbow flexion on dominant side	1.090	1.016–1.171	0.017	*
Age	1.994	1.294–3.074	0.002	*
Position: Pitcher compared with fielder	4.974	1.878–13.173	0.001	*

*ABER* ROM of 90°-abducted shoulder external rotation.

*P < 0.05.

### Univariate analyses post-hoc power analysis

In the univariate analyses, the post-hoc powers in elbow flexion, 90°-abducted shoulder external rotation, 90°-abducted shoulder internal rotation, shoulder total arc on the dominant side, shoulder supraspinatus strength ratio, ankle joint dorsiflexion and plantarflexion on the back leg, and plantarflexion on the front leg were 0.76, 0.73, 0.60, 1.00, 1.00, 0.81, 0.70, and 0.63, respectively.

### Sensitivity analysis of the logistic regression model

We performed a power analysis by applying the Monte Carlo method to test how the power of the model changed when each input variable was removed because it is difficult to completely avoid measurement error of input variables. We considered this analysis useful for separately interpreting each variable’s influence.

When ankle dorsiflexion on the back leg or 90°-abducted shoulder external rotation on the dominant side was removed, the powers substantially decreased (Table 4). However, when elbow flexion on the dominant side, age, or position (pitcher compared with fielder) were removed, each model had sufficient power. These results may indicate that ankle dorsiflexion on the back leg and 90°-abducted shoulder external rotation on the dominant side strongly contribute to the original model.

**Table 4:** Sensitivity analysis of a logistic regression model.

Deleted variable from the model	Power (%)
Ankle dorsiflexion on the back leg	49.7
Shoulder ABER on the dominant side	57.9
Elbow flexion on the dominant side	80.3
Age	97.9
Position: pitcher compared with fielder	86.7

## Discussion

We prospectively investigated whether ankle joint ROM affected the incidence of shoulder and elbow injuries in young baseball players after adjusting for differences in demographic data and upper extremity function. The most novel finding of this study was that an ankle joint dorsiflexion deficit on the back leg was a significant risk factor for shoulder and elbow injuries in young baseball players. In addition, we observed that older age, being a pitcher, increased elbow flexion, and decreased 90°-abducted shoulder external rotation were also significant risk factors for shoulder and elbow injuries. To our knowledge, this is the first prospective study to provide evidence that an ankle joint ROM deficit may be involved in shoulder and elbow injuries. This evidence may contribute to new strategies in injury prevention programs for baseball-related injuries.

A recent systematic review^30^ investigated the relationship between lower body function and elbow injury in baseball players and reported on several independent risk factors for elbow injuries in baseball players^8–21^. However, no study has investigated the relationship between ankle joint ROM and shoulder and elbow injuries in baseball players.

In our study, univariate analyses showed that ROMs of ankle joint dorsiflexion and plantarflexion on the back and front legs were significantly smaller in the injured group than in the non-injured group. Using logistic regression analysis to control for age, height, weight, all other ROM measures, and shoulder strength, a ROM deficit in ankle dorsiflexion on the back leg was identified as a risk factor for subsequent shoulder or elbow injuries. To interpret the effects of risk variation, we calculated the ORs of the conditions based on the mean differences between groups. If ankle dorsiflexion increased by 3.6°, the injury risk was reduced by 19% (calculated OR 0.81). Although the mean difference in ankle dorsiflexion on the back leg between the groups may appear small (3.6°), a 3.6° improvement can lead to an 19% reduction in the risk of injury, which is a substantial effect.

While this study cannot definitively determine why a ROM deficit in ankle dorsiflexion in the back leg increases the risk of injury, there are some potential explanations. First, as shown in a previous study involving the trunk and hip joint^30^, functional deficiencies in the ankle joint significantly affected kinetic changes in the adjacent segment or beyond. This finding supports the understanding that a transfer of force and energy occurs along the kinetic chain during the throwing motion^22,23,31^. If dysfunction occurs in one location (e.g., the ankle), an adverse effect occurs on other associated segments (e.g., knee, hip, trunk, shoulder, or elbow), presumably because the shoulder and elbow are forced to bear an increased load and force in an effort to overcome deficiencies at the ankle. When transferring the accumulated force into kinetic energy (i.e., from a wind-up phase to an arm-cocking phase) through loading the weight in the foot and ankle, the ankle on the back leg is required to dorsiflex appropriately. Therefore, a small limitation in ankle joint dorsiflexion in the back leg may have induced throwing-related injuries due to a significant loss of kinetic energy.

Furthermore, the dynamic balance may decrease because the ankle joint dorsiflexion ROM was reported to significantly influence dynamic balance^32,33^. Although it was possible because the dynamic balance was not evaluated in this study, if dysfunction in dynamic balance occurs, transmitted kinetic energy via the lower leg and trunk to the shoulder and elbow might be attenuated because kinetic energy may be used to compensate for the power necessary to stabilize the trunk. Consequently, the shoulder and elbow are presumably forced to bear an increased load to pitch. Although these explanations appear plausible, further studies are needed for validation.

In junior baseball players (aged 6–12 years), Sakata et al. showed that an elbow extension deficit ≥ 5° was significantly associated with medial elbow injury^16^. In high school baseball players, a glenohumeral internal rotation deficit (GIRD)^7^ in external rotation strength on the dominant side^7^ and weakness in the supraspinatus muscle on the dominant side^5^ were reported as significant risk factors. In professional baseball players, elbow varus angles and shoulder external rotation torque at peak external shoulder rotation during pitching^34^, GIRD^6^, shoulder flexion and external rotation insufficiency^8^, total shoulder rotation deficit^35^, preseason supraspinatus deficits, and prone external rotation strength weakness^4^ have been identified as risk factors for shoulder and elbow injuries.

Our findings indicated that ROMs in 90°-abducted shoulder external rotation and 90°-abducted shoulder internal rotation on the dominant side, and shoulder total arc on the dominant side were significantly smaller in the injured group than in the non-injured group, which is consistent with the results of previous studies. Using logistic regression analysis to control for age, height, weight, all other ROM measures, and shoulder strength, a 90°-abducted shoulder external rotation deficit on the dominant side was associated with an increased risk of subsequent shoulder or elbow injuries. Camp et al. reported that a preseason 90°-abducted shoulder external rotation deficit was an independent risk factor for elbow injuries during the upcoming season in professional baseball players^36^.

Regarding supraspinatus strength, Byram et al. revealed that supraspinatus weakness was significantly related to shoulder injuries in professional baseball pitchers^4^. In high school pitchers, Shitara et al. demonstrated that supraspinatus strength was not an independent risk factor for shoulder injuries^7^. In our study, although the supraspinatus strength ratio of the dominant side to the non-dominant side was significantly greater in the injured group than that in the non-injured group, it was not an independent risk factor for injuries. This may have been due to differences in performance levels and the maturity of young players. Whether the supraspinatus strength ratio is associated with injuries remains unclear, and further studies are required for clarification.

Werner et al. reported that greater elbow flexion was associated with an increase in ball velocity^37^. Aguinaldo et al. found that increased elbow flexion was related to an increased valgus torque, which might affect pitching-related elbow injury^38^. However, our findings indicated that elbow joint ROM in flexion on the dominant side was significantly greater in the injured group than in the non-injured group. Similarly, this may have been due to differences in performance levels and maturity among young players. Nevertheless, future analyses are warranted to determine the association between elbow flexion and injury.

In young baseball players, previous studies have reported that age ≥ 9 years was a significant risk factor for elbow injury^18,39^. Lyman et al. indicated that increased weight was a risk factor for elbow pain among skeletally immature baseball players^40^. Additionally, retrospective studies have shown that increased weight^41^ and height^41,42^ were risk factors for throwing-related shoulder and elbow pain.

Consistent with previous studies involving young baseball players, we found that participants’ age, height, and weight were significantly greater in the injured group than in the non-injured group. When performing logistic regression analysis, height and weight were removed from the explanatory variables because of the high correlation with age. Logistic regression analysis revealed that older age was a risk factor for injuries.

Corresponding with the results of previous studies ^3,39,40,43^ highlighting the risk for pitchers and catchers, our univariate analyses indicated that pitchers and catchers were significantly more likely to be in the injured group than in the non-injured group, and playing as a pitcher was a significant independent risk factor for shoulder and elbow pain using multivariate analyses.

This study had some limitations. First, it is difficult to compare this study with previous studies that include joint kinematics during throwing because joint dynamic ROM during flat-ground throwing or pitching was not measured in this study. Second, we did not investigate other potential risk factors in relation to hip and knee functions in the physical examination. As discussed, hip ROM deficits, abnormal arch posture, and lower extremity injuries were reported as independent risk factors for elbow injuries in baseball players. However, while no participants were unable to play baseball because of the trunk, hip, and knee injuries during the season, a prospective study on whole-body function is necessary in relation to pitching and throwing, and accurate analyses are needed. Third, we did not include the amount of external load in relation to throwing counts because these precise data were too difficult to obtain in our young baseball players, which may have affected our results. Fourth, our passive method of measuring ankle joint ROM, especially dorsiflexion, might have affected the results because the knee is slightly flexed, and the ankle is weight-bearing during the throwing motion. Finally, we did not collect data on injury severity; therefore, the severity level of injuries could not be determined. However, the injuries were unlikely to have been severe because no participants were found to have undergone shoulder or elbow surgery during the follow-up period. These limitations should be considered in future studies.

In young baseball players, an ankle dorsiflexion deficit in the back leg was a significant risk factor for shoulder and elbow injuries, as were increased age, being a pitcher, decreased shoulder abduction-external rotation on the dominant side, and increased elbow flexion on the dominant side. This evidence should be considered when designing injury prevention programs for baseball-related injuries.

## Methods

### Participants

Our department provides free annual preseason medical check-ups for young baseball players who belong to a prefecture baseball federation for the early detection of physical problems. In this prospective cohort study, we recruited young baseball players aged 7–12 years during preseason medical check-ups in 2017 and 2018 after explaining the study in detail. Informed consent for study participation and publication was obtained from the participants’ parents. The inclusion criteria included players who had: (1) participated in medical check-ups in both 2017 and 2018, (2) participated in preseason practice as an active player, and; (3) had no restrictions in baseball activities, including throwing, running, and batting^7,44^. The exclusion criteria were: (1) prior throwing arm injury and; (2) inability to play baseball due to foot, ankle, knee, hip, spine, shoulder, or elbow injuries^7,44^.

The Institutional Review Board of Gunma University Hospital (Identification number 1003) approved this study. All study procedures were performed in accordance with relevant guidelines and regulations.

### Medical check-ups

Preseason medical check-ups in 2017 were performed as baseline medical examinations to evaluate shoulder, elbow, and ankle conditions^7,44^. To avoid confirmation bias, the examiners were not informed of participants’ hand dominance. The following items were evaluated: height, weight, shoulder, elbow, and ankle joint ROMs, and shoulder muscle strength.

#### Shoulder, elbow, and ankle joint ROMs

The intra-rater validity and reliability of ROM measurements using a digital protractor have been previously established^7^. In our study, certified orthopedic surgeons bilaterally measured passive elbow joint ROM in flexion and extension, passive shoulder joint ROMs in shoulder horizontal adduction and 90°-abducted shoulder external and internal rotation, and passive ankle joint dorsiflexion and plantarflexion^7,44^ using a digital protractor (iGaging, Los Angeles, CA, USA). All ROMs were measured with participants in a supine position.

Similarly, passive ankle joint dorsiflexion and plantarflexion were measured with participants in the supine position with knee extended as previously described^45^. This position was selected because we aimed to measure the ankle at a position similar to the throwing motion, from the wind-up phase to the arm-cocking phase. To measure passive dorsiflexion, a digital protractor was aligned along the medial border of the tibia and the medial aspect of the foot. To measure passive plantarflexion, a digital protractor was aligned along the medial border of the tibia and along the medial aspect of the foot in line with the navicular tuberosity.

#### Shoulder strength

Intra-rater validity and reliability of shoulder strength measurements using hand-held dynamometers have been established in a previous study^7^. In our study, certified orthopedic surgeons used a PowerTrack II Commander hand-held dynamometer (J-Tech Medical, Salt Lake City, UT, USA). Participants were seated for the measurement of supraspinatus muscle strength and were subsequently positioned prone to measure prone internal rotation and prone external rotation strengths in both shoulders, in accordance with previous studies^7,44^. The dominant to non-dominant ratios of supraspinatus, prone external rotation and prone internal rotation strengths, and the prone external rotation-to-prone internal rotation strength ratio in the dominant arm were calculated for each participant.

### Injury tracking during the 2017 season

A shoulder or elbow injury (i.e., “Injured”) was defined as inability to throw for ≥ 8 days because of an elbow/shoulder problem^7,44,46^. Injuries that occurred due to other mechanisms, such as being hit by a ball, colliding with another player, or trauma from a fall, were excluded from the statistical analyses. To avoid recall bias, participants were asked to complete a daily self-recorded questionnaire regarding the presence of shoulder and/or elbow pain, limitations in pitching due to shoulder or elbow pain, and the presence of other injuries.

### Statistical analysis

A priori statistical power analysis for the logistic regression analysis indicated that a total of 70 participants would be needed to detect statistical significance depending on a statistical power of 80% at an α level of 0.05 (assumptive incidence rate, 20%; odds ratio [OR] 2.5)^6^.

All tests were two-sided with a significance level set at P = 0.05. Baseline characteristics and univariate analyses results are reported as means ± standard error. Group differences of baseline characteristics were evaluated using an independent t test or Mann–Whitney U test for continuous data and a chi-square test for categorical data. A residual analysis was performed to identify the specific cells making the greatest contribution to the chi-square test result. Finally, to test the hypothesis that limited ankle joint ROM was related to risk factors for shoulder and elbow injuries after adjusting for differences in demographic data and upper extremity function and to calculate ORs and 95% confidence intervals, a logistic regression analysis was performed to predict the outcome of the injury, which was used as a dependent variable. Independent variables considered for the model were selected based on the univariate analyses results (P < 0.1)^47^.

All statistical analyses were conducted using IBM SPSS version 23 (IBM Japan, Ltd, Tokyo, Japan, https://www.ibm.com/jp-ja/products/spss-statistics). Finally, a post-hoc power analysis was performed for the univariate analysis, and a sensitivity analysis of the logistic regression model was conducted to calculate power using the Monte Carlo method, with Stata 16 software (Stata Corporation, College Station, Texas USA, https://www.stata.com).

## Data Availability

The data supporting the findings of this study are available on request from the corresponding author, H.S. The data are not publicly available because they contain information that could compromise the privacy of research participants.
